# A Dictionary of Medical Science

**Published:** 1846-09

**Authors:** 


*A Dictionary of Medical Science.* By Robley Dungleson, M. D., Prof. of the Institutes of Medicine in the Jefferson Medical College, Philadelphia. Sixth edition. Philadelphia: Lea & Blanchard, 1846.

Both as a medical writer and teacher, Professor Dungleson ranks among the first in America. He has, perhaps, contributed more largely to the literature of medicine than any other one individual, and no better evidence is wanting of the high estimation in which the work before us is held, both by the student and medical practitioner, than is found in the fact that it has reached its sixth edition in about that number of years, and the superiority of the last, over all of the preceding ones, may be inferred from the fact, that it contains about twenty-five hundred words more than the fifth. We believe every term used in the various branches of medicine may be found in the present edition of Professor Dungleson's Dictionary. Most of the terms, too, used in dental surgery are contained in this work, so that it will be nearly, if not quite, be as useful to the dentist as to the physician. We can, therefore, commend it to the members of our own immediate branch of the profession.—*Balt. Ed.*

